# The Challenge of Targeting Notch in Hematologic Malignancies

**DOI:** 10.3389/fped.2014.00054

**Published:** 2014-06-10

**Authors:** Fiorela N. Hernandez Tejada, Jorge R. Galvez Silva, Patrick A. Zweidler-McKay

**Affiliations:** ^1^Department of Pediatrics, University of Texas M. D. Anderson Cancer Center, Houston, TX, USA

**Keywords:** Notch signaling, leukemia, lymphoma, oncogenes, tumor suppressor

## Abstract

Notch signaling can play oncogenic and tumor suppressor roles depending on cell type. Hematologic malignancies encompass a wide range of transformed cells, and consequently the roles of Notch are diverse in these diseases. For example Notch is a potent T-cell oncogene, with >50% of T-cell acute lymphoblastic leukemia (T-ALL) cases carry activating mutations in the Notch1 receptor. Targeting Notch signaling in T-ALL with gamma-secretase inhibitors, which prevent Notch receptor activation, has shown pre-clinical activity, and is under evaluation clinically. In contrast, Notch signaling inhibits acute myeloblastic leukemia growth and survival, and although targeting Notch signaling in AML with Notch activators appears to have pre-clinical activity, no Notch agonists are clinically available at this time. As such, despite accumulating evidence about the biology of Notch signaling in different hematologic cancers, which provide compelling clinical promise, we are only beginning to target this pathway clinically, either on or off. In this review, we will summarize the evidence for oncogenic and tumor suppressor roles of Notch in a wide range of leukemias and lymphomas, and describe therapeutic opportunities for now and the future.

## Introduction

“Notch” is a critical developmental pathway, which controls cell fate, differentiation, proliferation, and survival, and is critical in numerous developmental processes including neurogenesis, angiogenesis, and hematopoiesis, among others (1). The ability of Notch signaling to inhibit or induce differentiation, drive or impair proliferation, and promote survival or induce apoptosis in a cell-specific manner provides the opportunity for Notch signaling to contribute to or impede oncogenesis in multiple cell types (2).

The role of Notch has been studied in a wide variety of hematological malignancies including T and B leukemias and lymphomas as well as myeloid leukemias (3–5). However, the consequences of Notch signaling in these diseases varies significantly, and thus targeting Notch, requires understanding the biology of Notch signaling on a disease-by-disease basis (Table 1).

**Table 1:** **The Notch pathway by cancer type**.

	Oncogene vs. tumor suppressor	Genetic lesions	Evidence
**LYMPHOID CANCERS**			
T-cell acute lymphoblastic leukemia	Oncogene	>50% Notch1 HD/PEST activating mutations, 15% FBXW7 mutations	T-cell oncogene in mice, Notch inhibition impairs T-ALL growth and survival. Some clinical responses to Notch inhibitors.
T-cell non-Hodgkin lymphoma	Oncogene	50% Notch1 HD/PEST activating mutations, 20% FBXW7 mutations	Notch inhibition induces apoptosis
B-cell acute lymphoblastic leukemia	Tumor suppressor	Methylation of Notch target genes, no activating mutations	Notch activation impairs B-ALL growth and survival
Chronic lymphocytic leukemia	Oncogene	5–15% Notch1 PEST activating mutations	Notch inhibition induces apoptosis
Hodgkin lymphoma	Oncogene	none	Notch activation induces growth and survival
B-cell non-Hodgkin lymphoma	Oncogene^a^	5–20% Notch1/2 PEST activating mutations	Notch inhibition impairs growth but may promote survival in some
**MYELOID CANCERS**			
Chronic myelomonocytic leukemia	Tumor suppressor	12% Notch pathway inhibiting mutations	Notch loss induces CMML-like disease in mice
Acute myeloblastic leukemia	Tumor suppressor^a^	Methylation of Notch target genes, no activating mutations	Notch activation impairs AML growth and survival, Notch inhibition promotes AML *in vivo*
Chronic myelocytic leukemia	Unclear	None	Notch aids blast crisis in mice, but is decreased in humans. Notch activation impairs CML growth and survival

*T-ALL, T-cell acute lymphoblastic leukemia; B-ALL, B-cell acute lymphoblastic leukemia; CMML, chronic myelomonocytic leukemia; AML, acute myeloid leukemia; HD, heterodimerization domain; PEST, proline–glutamine–serine–threonine-rich; FBXW7, F-box/WD repeat-containing protein 7; CML, chronic myelocytic leukemia*.

*^a^Contrasting evidence*.

In this review, we will outline the roles of the Notch pathway in a wide variety of leukemias and lymphomas, describe potential targeted therapies and discuss future directions.

## Notch Pathway

In mammals, the Notch signaling pathway consists of five cell membrane-based ligands [Jagged1, Jagged2, Delta-like ligand-1 (DLL1), DLL3, and DLL4], each of which bind to and activate four cell membrane-based Notch receptors (Notch1–4) present on neighboring cells. Receptor interaction with ligand classically requires cell–cell interaction and induces enzymatic cleavage of the Notch receptors at their transmembrane domain by both metalloproteinases and the gamma-secretase complex (6). This releases the intracellular domain of Notch (ICN) from the membrane, which then translocates into the nucleus and associates with a common transcription factor RBPjk (also known as CSL), leading to the expression of Notch target genes, e.g., the HES/HEY family, which can vary depending on cell type (7). Thus, Notch signaling can occur in a variety of circumstances based on the presence of the five different ligands in the microenvironment, expression of metalloproteinase, and gamma-secretase complex enzymes, and the expression of the four Notch receptors, yielding a large number of potential variations on the Notch pathway.

## Notch in Lymphoid Leukemias and Lymphomas

The normal developmental roles of the Notch pathway in lymphopoiesis have led to both similarities and contrasting findings across the range of lymphoid malignancies. For example, Notch has well-defined roles in inducing commitment, differentiation, and function in the T-cell lineage, while impairing early B-cell development, and inducing more mature marginal zone B-cell differentiation. Thus, in normal development Notch appears to generally support T-cell growth, differentiation, and survival, while effects in B cells appear to depend on the stage of differentiation, inhibiting immature B cells, and supporting at least a subset of more mature B cells (8).

### T-cell acute lymphoblastic leukemia

Aberrant Notch activation was first linked to cancer in 1991 when Notch1 was identified as part of a t(7:9)(q34;q34) translocation in patients with T-cell acute lymphoblastic leukemia (T-ALL) (9). This translocation leads to high levels of truncated, constitutively active intracellular Notch1 (ICN1), implicating Notch as a T-cell oncogene. However, this translocation was subsequently found in <1% of human T-ALL. This discovery however initiated numerous studies of Notch signaling in normal T-cell development and leukemogenesis, where Notch was found to be a critical T-cell pathway and a potent T-cell oncogene in mice (10, 11). Over a decade later, it was shown that >50% of T-ALL cases have activating mutations in the Notch1 gene, revealing Notch1 mutation as the most common oncogenic lesion in T-ALL (12). Activation of Notch1 is found in all subtypes of T-ALL, including TAL1, HOX11, HOX11L2, LYL1, MLL-ENL, and AF10-CALM, suggesting that Notch1 activation is a fundamental event in T-cell transformation. Interestingly, two major mutational hot spots were characterized in T-ALL, with missense mutations in the heterodimerization (HD) domain in 30–45% of T-ALL cases, leading to lower protection from cleavage of Notch, resulting in potent Notch activation, and nonsense or missense mutations in the proline–glutamate–serine–threonine-rich (PEST) degradation domain in 20–25% of T-ALL, allowing for prolonged but less potent Notch signaling. In addition, Notch signaling is aberrantly activated through mutation of the Notch1-targeting E3 ligase FBXW7 in 10–15% of T-ALL cases, which leads to prolonged Notch activation through a similar mechanism to PEST domain mutation (13, 14). The relative strength of Notch signaling induced by the different mutations may provide insight into oncogenic mechanisms and may potentially be useful in selecting patients for Notch inhibitor therapies.

In patients with T-ALL, Notch1 and FBXW7 mutations have generally been associated with favorable prognosis and lower minimal residual disease (MRD) levels (15–17). However, Notch mutations have also been associated with higher rates of CNS relapse and poor outcome in Notch mutated patients with high end-induction MRD (18). Another study suggested that Notch/FBXW7 mutations predict better outcome only in the absence of RAS or PTEN alterations (19). Interestingly, Notch1 mutations occur at lower frequency in early thymic progenitor (ETP) T-ALL (20), suggesting alternate mechanisms of Notch activation, or distinct leukemogenic mechanisms. Given the overall high frequency of Notch activating lesions, inhibiting Notch in T-ALL has been an attractive therapeutic opportunity.

### T-cell non-Hodgkin lymphoma

Similar to T-ALL, T-NHL can carry activating Notch1 and/or inhibiting FBXW7 mutations in up to 50% (7/14) of cases (21), though the frequency may be lower in more mature T-NHL (22). Interestingly Notch1 is frequently expressed in anaplastic large cell lymphoma (ALCL) cells (23), and Notch signaling appears to induce proliferation and survival (24).

### B-cell acute lymphoblastic leukemia

The role of Notch signaling in B-cell acute lymphoblastic leukemia (B-ALL) is less clear. In B-ALL, a disease of immature B-precursor cells, no Notch mutations have been found, and although Notch receptors are expressed, Notch signaling does not appear to be constitutively activated, in contrast to T-ALL (25, 26). Methylation studies reveal that several of the Notch pathway target genes are silenced in B-ALL, and re-expression inhibits growth and survival (27). Several studies have shown that induced activation of the Notch pathway in human B-ALL cells leads to growth arrest and apoptosis, suggesting a tumor suppressor role for Notch (26–28). In contrast, another report suggests that Notch signaling supports B-ALL in the bone marrow niche (29). Although the roles for Notch signaling in B-ALL are not yet fully defined, there may be an opportunity to pharmacologically induce Notch signaling in B-precursor ALL as a therapeutic approach.

### Chronic lymphocytic leukemia

Chronic lymphocytic leukemia is a disease of mature B cells, which occurs in older adults. Recent sequencing data revealed that 5–15% of CLL samples carry apparent activating mutations in Notch1 (30–33). These were enriched in chemo-refractory disease (21%) and in patients whose CLL had undergone Richter’s transformation (31%) (33). These mutations occur almost exclusively in the PEST domain of Notch1, not the HD domain as seen in T-ALL, and >80% are a single recurrent 2-bp deletion (7544_7545 delCT), suggesting a related but distinct mutational profile to Notch1 mutations in T-ALL. *In vitro*, Notch signaling appears to prevent CLL apoptosis (34). In patients, Notch1 mutation is associated with poor outcome and resistance to fludarabine treatment (30, 31, 35, 36).

### Hodgkin lymphoma

Expression of Notch1 and ligand Jagged1 were shown in classical HL Reed-Sternberg cells (37). Activation of Notch signaling *in vitro* induced proliferation and survival in HL cells (24). Conversely, Notch inhibition led to decrease in NF-kB activity, supporting an oncogenic role for Notch in HL (38). Interestingly, it has been suggested that Notch signaling in HL leads to the loss of B-cell markers through repression of critical B-cell genes E2A and EBF (39).

### B-cell non-Hodgkin lymphoma

In B-cell NHL, evidence for Notch activation occurs in a subset of lymphoma subtypes. Mutations are found either in Notch1 or Notch2, and occur in the PEST domain, but not the HD domain, similar to CLL, but in contrast to T-ALL. In typically MYC-driven Burkitt lymphoma, 7% (5/70) carry Notch1 mutations (40), 8% (5/63) of BCL2-associated diffuse large B-cell lymphoma (DLBCL) carry similar PEST mutations of Notch2, and 6% (2/35) had amplification of the Notch2 locus (41). Marginal zone lymphomas also carry 5% (2/41) to 20% mutated Notch2, in addition to rare Notch1, SPEN, and DTX1 mutations (42, 43). Finally, >12% Notch1 mutations were found in mantle cell lymphomas and were associated with poor survival (44). Notably, Notch activating mutations have not been found in B-cell lymphoblastic and follicular lymphomas. These studies reveal that subsets of several mature B-NHLs carry Notch1/2 PEST mutations, suggesting an oncogenic role for Notch in these cancers.

## Notch in Myeloid Leukemias

In myeloid cells, Notch may have a range of effects including inhibiting or promoting differentiation and stimulating or impairing growth and survival, depending on the cell type studied. Importantly, genetic inhibition of Notch signaling in murine models can lead to increased myeloid cells and myeloproliferation, suggesting that Notch may generally inhibit myeloid development (45–47). However, the roles of Notch in different myeloid leukemias have not been fully characterized.

### Chronic myelomonocytic leukemia

Chronic myelomonocytic leukemia is a rare myeloproliferative and myelodysplastic leukemia, which typically occurs in older adults. However, a recent study found that inactivation of Notch signaling in murine bone marrow led to a myeloproliferative disease, and identified inactivating mutations in Notch pathway genes (NCSTN, APH1, MAML1, and NOTCH2) in 12% (5/42) CMML patient samples, implicating a tumor suppressor role for Notch in this disease (48).

### Acute myeloblastic leukemia

With the unclear roles of Notch in myelopoiesis, murine models were used to investigate whether loss of Notch would alter myeloid leukemogenesis. Indeed loss of Notch in combination with loss of the myeloid tumor suppressor TET2 led to an AML-like disease in mice, suggesting a formal tumor suppressor role for Notch in AML (49). Consistent with this, human AML samples do not carry activating mutations in Notch pathway genes, except in rare cases of recurrent T-myeloid leukemias, which can carry Notch1 activating mutations from the initial T-ALL. AML cells do express Notch receptors on their surface, however, they lack constitutive Notch signaling and demonstrate methylation Notch pathway genes, similar to B-ALL (25, 49). In some studies, activation of Notch signaling in AML cells led to growth arrest, apoptosis, and differentiation, while inhibition of Notch led to increased aggressiveness *in vivo*, suggesting a tumor suppressing effect in AML (49–51). The mechanism of this effect may involve Notch-mediated suppression of CEBPA, Pu.1, BCL2, and the stabilization of p53 expression. In contrast, in some studies Notch signaling has variable effects on AML growth and survival, depending on the individual AML sample (52). Finally, Notch1, Jagged1, and DLL1 expression in patient samples were associated with poor outcome though the activity of the Notch pathway was not measured (53). Thus, there is generally evidence for a tumor suppressing effect in AML, however additional studies are needed to better characterize the roles of Notch in AML.

### Chronic myelogenous leukemia

Chronic myelogenous leukemia is a mature myeloproliferative disease driven by the expression of BCR-ABL1 as a consequence of *t*(9;22) translocation. BCR-ABL1 appears to enhance Notch1 expression and activation, leading to decreased MYC expression and colony formation (54). In a murine model, expression of the Notch target gene HES1 was shown to cooperate with BCR-ABL1 expression to promote CML blast crisis (55). In contrast, HES1 downregulation was associated with blast crisis in human patient samples (56). Induced activation of Notch signaling in CML-derived cell lines reveal growth inhibition, suggesting an inhibiting role for Notch in CML (50, 57, 58). Thus, murine leukemogenesis studies suggest an oncogenic role while human studies suggest a tumor suppressor role, leaving the role of Notch in CML unclear.

## Notch Targeting Therapeutics

**Figure 1:**
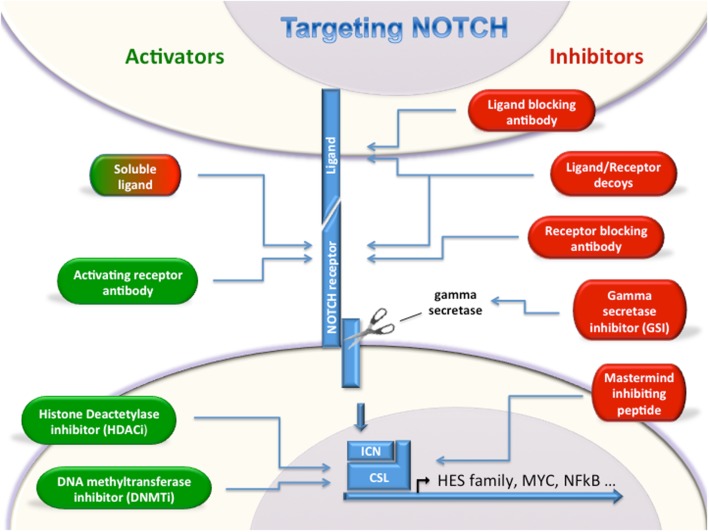
**Targeting Notch**. Multiple strategies for either activating or inhibiting Notch have been described. Notch activators (green, left) and Notch inhibitors (red, right) will allow modulation of Notch depending on oncogenic or tumor suppressor roles in a given cancer type. Soluble ligands can either activate or inhibit Notch signaling.

Given the emerging data demonstrating roles for Notch signaling in a wide variety of leukemias and lymphomas, targeting the Notch pathway either with inhibitors or activators is a very compelling therapeutic possibility. However, caution should be used when targeting this pathway as disease-specific responses to Notch modulation may be counter-therapeutic. Several Notch inhibiting strategies are being tested in clinical trials, while Notch activators are still in the early pre-clinical stage (Figure 1).

## Notch Inhibiting Strategies

### Gamma-secretase inhibitors

Interaction between Notch ligands and receptors induces a conformational change in the Notch receptors’ HD domain, which allows for enzymatic access to the transmembrane region. Following ligand interaction, the receptors are cleaved by ADAM/TACE metalloproteinases, and subsequently cleaved again by the gamma-secretase complex, which is a crucial step in the activation of Notch signaling. It is this step, which is targeted by GSIs [reviewed in Ref. (59)].

The potent role of Notch signaling in T-cell leukemogenesis and >50% presence of activating Notch1 gene mutation in T-ALL has prompted the testing of GSIs in multiple clinical trials, though most results are unpublished, BMS-906024 (NCT01363817), RO4929097 (NCT01088763), MK0752 [NCT00100152 (60)], PF03084014 (NCT00878189), and LY3039478 (NCT01158404). However, early trials were hampered by excessive toxicity from on-target effects on the intestinal epithelial differentiation, resulting in dose-limiting diarrhea (60, 61). Alternative schedules and dosing have been sought to ameliorate these symptoms with some success. One attractive combination is the use of glucocorticoids and GSI. In a murine study, it was demonstrated that steroids can ameliorate the GSI-induced gut toxicity *in vivo*, protecting the animals from developing intestinal goblet cell metaplasia (62). In addition, GSI treatment induces glucocorticoid receptor expression and restores steroid sensitivity (63). As single agent Phase I trials continue to address toxicity and activity, multiple mechanisms of resistance have been identified. For example, PTEN loss has been associated with GSI resistance (64). Also, GSI resistant cells appear to have distinct epigenetic status, and BRD4-inhibiting JQ1 has been shown to synergize with GSI (65). For future trials several classes of agents, including PI3K/mTOR, histone deacetylase, and proteasome inhibition, have been shown to enhance GSI effects in T-ALL (66–68).

The use of GSI in T-cell NHL, including ALK+ ALCL, has shown some pre-clinical promise inducing growth inhibition and caspase-mediated apoptosis with downregulation of cyclin D1, Bcl-XL, and XIAP (23, 69). Similarly, GSIs show pre-clinical promise for CLL, demonstrating decreased NF-kB, XIAP, c-IAP2 levels (34), though some data suggest Notch-independent mechanisms (70). GSIs have also been effective in two mantle cell lymphoma lines (44) and one DLBCL line without Notch mutations (71).

In contrast, pre-clinical testing of GSIs in some B-NHLs has shown a lack of efficacy and even perhaps pro-survival effects (72). And while some data suggest pre-clinical efficacy of GSIs in AML (73), other studies found that GSIs unexpectedly increased HES1 expression in GSI-sensitive B-NHL and AML lines (74), calling into question the rationale and mechanism for GSI effects in these diseases.

### Monoclonal antibodies

Another class of agents under development is the mAb targeted against Notch receptors or ligands, or the gamma-secretase complex. The anti-receptor antibodies inhibit the production of cleaved “activated” Notch receptors, e.g., Notch1 OMP52M51 (75), Notch2 OMP-59R5, Aveo anti-Notch1 or Notch3 (76), and Genentech anti-Notch1 or Notch2 (77). Experimental evidence demonstrates that Notch inhibition by either mAb against Notch1 or Notch2 appears to have anti-tumor and anti-angiogenic effects with limited gastrointestinal toxicities while simultaneous inhibition of Notch1 and 2 lead to gastrointestinal toxicity, as seen with many GSIs (77–79). Anti-ligand antibodies targeting DLL4 (REGN421, OMP-21M18), which block the ability of ligand to activate Notch receptors, have been shown to induce disorganized angiogenesis, reduce perfusion, and impair solid tumor growth while sparing intestinal toxicities *in vivo* (80, 81). Finally, an antibody against the gamma-secretase complex (A5226A) has shown pre-clinical activity against T-ALL (82).

It is hoped that this category of drugs could reduce or spare some of the toxicities associated with pan-Notch inhibition by GSIs, though this has not yet been confirmed clinically. These antibodies have not all been tested in hematologic cancers.

### Decoys

Additional approaches to inhibit Notch signaling come from the use of proteins, fragments, or peptides, which inhibit Notch signaling. First, soluble Notch pathway proteins have been shown to inhibit Notch signaling through saturation of the Notch receptors with soluble ligand DLL4-Fc (81, 83), Jagged1 (84), DLK1 (85), EGFL7 (86), or through binding of ligands through soluble Notch1 receptor extracellular domain (87). Another decoy approach that has been developed is a Mastermind inhibiting peptide, which mimics the critical interaction domain of Mastermind-like1 (MAML1) blocking the interaction of MAML with the Notch intracellular domain (88). The success of these therapies may rely on the specific biology of a given tumor and the breadth and potency of Notch inhibition achieved. Although, these approaches may provide more options to inhibit Notch signaling, their protein/peptide structure makes them somewhat difficult to transform into a reliable clinical therapeutics.

## Notch Activating Strategies

### Ligand-mimicking proteins/peptides

Soluble Notch ligands are generally thought to inhibit Notch receptor cleavage, however, several studies have demonstrated the feasibility of using such Notch ligands and ligand-mimicking proteins as agonists, e.g., clustered DLL1 extracellular domains (89), DLL1 DSL domain (90), Jagged1 DSL peptide (50, 91, 92), DNER (93), TSP2 (94), CCN3 (95), YB-1 (96), NB-3 (97), and periostin (98). Although, all of these proteins have been shown to activate Notch signaling in at least one model system, the role of most of these Notch agonists in cancer remains to be evaluated. Importantly, a Jagged1 DSL peptide has been shown to be effective *in vitro* against B-ALL (28) and AML (50), suggesting potential of Notch agonists as cancer therapeutics depending on tumor type.

### Notch receptor activating antibodies

Monoclonal antibodies have been developed, which are capable of specifically inducing cleavage of the Notch2 and Notch3 receptors (99, 100). These activating antibodies have the advantage of selectively inducing cleavage of one of the Notch receptors, allowing one to choose the best target in a given disease and avoiding global Notch activation, when desired. Notch receptor activating antibodies have not been evaluated in cancer models.

### Inducers of Notch

In addition to targeted agents, which induce Notch signaling through Notch ligands or receptors, epigenetic modifiers, e.g., histone deacetylase inhibitors and DNA methyltransferase inhibitors, can induce Notch signaling in cancers where Notch plays a tumor suppressor role (27, 101–103).

## Future Directions

Currently, several different GSIs and Notch inhibiting antibodies are in clinical trials, with additional Notch inhibiting approaches in near clinical development. With these agents, our greatest challenges are to overcome the intestinal toxicity caused by continuous Notch inhibition, identify patients who are likely to respond to Notch inhibition, and determine what combinations hold the most promise for diseases where the oncogenic role of Notch is fairly clear, i.e., T-ALL, CLL, and T-NHL, some mature B-NHL, perhaps HL. Looking toward the future, we hope to have Notch activators in clinical trial in the next few years so that we may target Notch in diseases where Notch is likely a tumor suppressor, i.e., CMML, AML, and B-ALL.

The Notch pathway is complex and the wide range of consequences in different cancer subtypes makes targeting this pathway challenging. However, as we learn more about the mechanisms and consequences of Notch signaling in the range of leukemia and lymphoma subtypes, we will be able to target Notch signaling to specifically impair the growth, survival, and/or differentiation of that disease while reducing the toxicities. With a growing number of Notch modulating therapeutic agents, we may have the tools to customize Notch targeting in the near future.

## Conflict of Interest Statement

The authors declare that the research was conducted in the absence of any commercial or financial relationships that could be construed as a potential conflict of interest.
